# Effect of clock rhythm on emergence agitation and early postoperative delirium in older adults undergoing thoracoscopic lung cancer surgery: protocol for a prospective, observational, cohort study

**DOI:** 10.1186/s12877-024-04846-0

**Published:** 2024-03-12

**Authors:** Linghui Jiang, Jie Wang, Wannan Chen, Zhiyao Wang, Wanxia Xiong

**Affiliations:** Department of Anesthesiology, Zhongshan Hospital, Fudan University, 200032 Shanghai, China

**Keywords:** Clock rhythm, Emergence agitation, Early postoperative delirium, Older adults

## Abstract

**Introduction:**

Surgeries conducted at night can impact patients’ prognosis, and the mechanism may be related to circadian rhythm, which influence normal physiological functions and pathophysiological changes. Melatonin is primarily a circadian hormone with hypnotic and chronobiotic effects, thereby affecting disease outcomes through influencing the expression of inflammatory factors and biochemical metabolism. This study aims to observe the effects of circadian rhythms on emergence agitation and early postoperative delirium of older individuals undergoing thoracoscopic lung cancer surgery and explore the possible regulatory role of melatonin.

**Methods:**

This prospective, observational, cohort study will involve 240 patients. Patients will be routinely divided into three groups based on the time of the surgery: T1 (8:00–14:00), T2 (14:00–20:00) and T3 group (20:00–08:00). The primary outcome will be the incidence of emergence agitation assessed via the Richmond Agitation and Sedation Scale (RASS) in the post-anesthesia care unit (PACU). Secondary outcomes will include the incidence of early postoperative delirium assessed via the Confusion Assessment Method (CAM) on postoperative day 1, pain status assessed via the numerical rating scale (NRS) in the PACU, sleep quality on postoperative day 1 and changes in perioperative plasma melatonin, clock genes and inflammatory factor levels. Postoperative surgical complications, intensive care unit admission and hospital length of stay will also be evaluated.

**Discussion:**

This paper describes a protocol for investigating the effects of circadian rhythms on emergence agitation and early postoperative delirium of older individuals undergoing thoracoscopic lung cancer surgery, as well as exploring the potential regulatory role of melatonin. By elucidating the mechanism by which circadian rhythms impact postoperative recovery, we aim to develop a new approach for achieving rapid recovery during perioperative period.

**Trial registration:**

The study was registered at the Chinese Clinical Trials Registry (ChiCTR2000040252) on November 26, 2020, and refreshed on September 4, 2022.

## Background

Due to the increasing number of surgeries performed at large medical institutions, many elective surgeries are scheduled at night [1]. Studies have shown that undergoing surgeries at different times of the day can affect patients’ prognoses, with the mechanism potentially related to circadian rhythms [2–4]. Circadian rhythms, also known as clock rhythms, play a crucial role in physiological processes such as hormone secretion, inflammatory factor expression and biochemical metabolism in the human body, influencing normal physiological functions and pathophysiological changes [5, 6]. Sleep disorders and sleep deprivation can interfere with the expression of clock genes and cause circadian rhythm disorders [7]. Research have confirmed that sleep deprivation can lead to abnormal expression of brain and muscle Arnt-like protein 1(BMAL1), period circadian regulator 2 (PER2), and other clock genes in rat hippocampus, impairing hippocampal learning and memory. Additionally, sleep deprivation can increase the incidence of postoperative cognitive dysfunction [8]. Clinical and laboratory data have demonstrated that applying time-based therapeutics to formulate a rational administration plan can provide significant clinical value in improving drug efficacy and reducing adverse effects [9–11]. Wright et al. found that anesthesia complications differ when anesthesia is induced at different times on the same day, which may be related to different hormone levels and inflammatory factor expressions in patients [12]. Melatonin, a chronobiological hormone, secreted by the pineal gland, has a circadian rhythm, gradually increasing at night, peaking around dawn, and returning to baseline levels during the day [13, 14]. Melatonin connects external day-night alternations with physiological functions in the human body through hormone secretion, inflammatory factors, and biochemical metabolism, thereby influencing disease outcomes [15]. Recent studies showed the effects of melatonin premedication on perioperative sedation, orientation, anxiety scores and psychomotor performance [16–19]. Guidelines for Enhanced Recovery After Surgery indicate that improving perioperative rehabilitation quality in patients under multimodal interventions is gaining increasing recognition [20, 21]. By elucidating the mechanisms by which clock rhythms influence postoperative recovery, we can develop perioperative interventions that can reduce postoperative complications, shorten hospital stays and reduce the waste of medical resources [22–25].

The incidence of post-general anesthesia agitation is increasing, yet it is often unrecognized and untreated [26]. Delays in patient recovery can lead to inefficiencies in the post-anesthesia care unit (PACU), resulting in bottlenecks in patient flow. Furthermore, emergence agitation may indicate the presence of hyperactive delirium, and postoperative delirium is associated with longer hospital stays, increased morbidity and mortality and need for hospitalization [27]. Prospective observational studies have shown increasing incidences of emergence agitation ranging from 4.7 to 22.2%, especially occurring at night [28]. Different surgery times have been shown to affect the occurrence of perioperative adverse events and increase postoperative complications and mortality; however, few studies have examined the effects of different surgery times on agitation, particularly in the PACU. In this study, we will investigate whether there are variations in the primary outcome of emergence agitation and secondary outcomes including early postoperative delirium, pain status, sleep quality, melatonin levels and inflammatory factors differ during different time periods throughout the day. We aim to explore the possible regulatory role of melatonin to seek new methods for rapid recovery during the perioperative period.

## Methods

### Study design

This is a prospective, observational, cohort study. We will include 240 patients scheduled for elective surgery under general anesthesia at two centers: Zhongshan Hospital, Shanghai, China, and Zhongshan Hospital Xiamen Branch, Fudan University, Xiamen, Fujian, China. Patients will be divided into three groups by operation time: T1 (8:00–14:00), T2 (14:00–20:00) and T3 (20:00–08:00). Pre-experiments began in January 2021. Formal research began in September 2022 and will be completed in December 2023.

### Patient and public involvement

Neither patients nor the public will be involved in the design, conduct, reporting, or dissemination plans of our research.

### Sample size calculation

This study will test whether incidence of agitation in groups T2 and T3 will differ significantly from that in group T1 (the control group). A two-sided significance level of 0.025 (with Bonferroni’s adjustment for multiple comparisons) will be used for both comparisons. The power of each test is 0.8. According to the PACU agitation analysis results from July 2020 to Dec 2020 at our center, the agitation incidences by group were 0.012 for T1, 0.145 for T2 and 0.252 for T3. The sample size of each group is 73. Considering a 10% expulsion percentage, 80 patients are needed per group, yielding a total sample size of 240.With daily operation volumes of 180 at Zhongshan Hospital and 30 at Xiamen Hospital, 206 patients will be enrolled at Zhongshan Hospital, Fudan University, Shanghai, and 34 will be enrolled at Xiamen Hospital.

### Participants and groupings

Physicians will provide detailed explanations of the research and provide informed consent forms to eligible patients. Patients will have at least 24 h to fully consider whether to participate in this study. When patients agree to participate, they will sign informed consent forms, and the corresponding date will be recorded. Surgeons will schedule the surgeries routinely, and the researchers will assign patients to one of three groups according to operation time: T1 (8:00–14:00), T2 (14:00–20:00) or T3 (20:00–08:00).

### Eligibility criteria

#### Inclusion criteria

- Aged 65–80 years, men or women.
- Body mass index (BMI) 18.5–23.9.
- Undergoing elective thoracoscopic surgery for lung cancer.
- American Society of Anesthesiologists (ASA) physical statuses I–II.
- Able to communicate and sign the informed consent.

#### Exclusion criteria

- Urgent surgery.
- Pre-existing neuroendocrine or immune system diseases.
- Cardiac insufficiency with left ventricular ejection fraction < 40%.
- Severe hepatic insufficiency (prothrombin ratio < 15%).
- Severe renal insufficiency (eGFR < 30 ml/min/1.73m^2^).
- Pre-existing mental illness, refusal to participate or failure to sign the informed consent.

#### Withdrawal criteria

- Patients with severe intraoperative complications (e.g., bleeding, emphysema mediastinum, pneumothorax).
- Withdrawal of informed consent by participants.
- Need to terminate the study as medically evaluated by the researchers.

### Drugs

Propofol (50 mL:0.5 g, AstraZeneca S.p.A., England), remifentanil (1 mg, Humanwell Healthcare Co., Ltd., China), rocuronium (5 mL:50 mg, Organon, Netherlands), sevoflurane (Hengrui Pharmaceutical Co., Ltd., China), sufentanil (1 mL:50 µg, Yichang Renfu Pharmaceutical Industry, Hubei, China), oxycodone (1 mL:10 mg, Hamol Lid., England), and flurbiprofen axetil (5 mL:50 mg, Tide Pharmaceutical Co., Ltd., China) will be administered during and/or after surgery. Dexmedetomidine (2 mL:0.2 g, Yangtze River Pharmaceutical Co., Ltd., China) will be administered, if necessary, after surgery.

### Procedure

Except for the different operation time, all anesthesia techniques and surgical procedures for the three groups will be in accordance with the unified standard. One day before the operation, participants’ demographic data and medical history will be recorded. Preoperative sleep quality will be assessed via the Richards Campbell Sleep Questionnaire (RCSQ).

After entering the operating room, peripheral venous access will be established. Blood sample for gas analysis and melatonin, BMAL1, PER2, interleukin-6 (IL − 6), tumor necrosis factor-alpha (TNF − A), C-reactive protein (CRP), and procalcitonin (PCT) values will be collected before the induction of anesthesia. All participants will undergo preoxygenation (5 L·min^− 1^) through a face mask, and standard monitoring, including a five-lead electrocardiogram, non-invasive blood pressure, pulse oxygen saturation (O_2_) and end-tidal carbon dioxide (EtCO_2_) will be applied throughout the procedure. The mean arterial pressure will be maintained at 60–80 mmHg.

Venous blood will be extracted from patients twice before and after surgery, then immediately centrifuged. Serum will be collected and stored in a freezer at − 18 °C, then sent to Shanghai Yueyang Biological Engineering Co., Ltd. for serum melatonin level detection in batches. The remaining samples will be destroyed as biological waste.

#### Intravenous induction of general anesthesia

The drugs used to induce anesthesia will be the same for all participants: sufentanil (0.2 µg·kg^− 1^), propofol (2 mg·kg^− 1^), remifentanil (1 µg·kg^− 1^), rocuronium (0.6 mg·kg^− 1^).

#### Maintenance of general anesthesia

All patients will receive sevoflurane (with 50–70% inhaled oxygen concentration, 1 L·min^− 1^ gas flow and minimum alveolar concentration maintained at 0.8), oxycodone (0.1 mg·kg^− 1^), rocuronium (intermittent injection), and sufentanil (intermittent injection). Single-lung ventilation will be set to FiO_2_ 80%, Vt 6 mL/kg, f 12–14 per min, EtCO_2_ maintained at 35-45mmHg, and airway pressure ≤ 30 mmHg. Depth of anesthesia (BIS, Covidien, France) will be monitored. The bispectral index target will range between 40 and 60.

Postoperative protocol.

#### Extubation after verification of standard criteria

Spontaneous breathing with total expired volume ≥ 5–8 mL/kg, respiratory rate of 12–25 c/min, absence of residual curarization defined by T4/T1 ≥ 90% (train-of-four), SpO_2_ ≥ 95% with FiO_2_ ≤ 50%, verbal and motor response to simple orders, and body temperature ≥ 36 °C.

#### Postoperative treatment

Patients will be comprehensively evaluated using the Richmond Agitation and Sedation Scale (RASS) and numerical rating scale (NRS) after tracheal extubation every 10 min in the PACU. Early postoperative delirium will be evaluated on postoperative day 1 via the Confusion Assessment Method (CAM). Sleep quality will be assessed via the RCSQ on postoperative day 1.

Peripheral blood will be extracted to measure melatonin levels, BMAL1, PER2 and arterial blood gases again in the PACU.

For patients with RASS scores ≥ 2, the following observations will be noted:

(1) The need for additional staff to handle and pacify agitated patients.

(2) Fall from bed or operating table.

(3) Unintentional discontinuation of drainage tubes/intravenous access or disruption of cicatrices due to patients’ agitation.

For patients with RASS scores ≥ 2 or NRS scores ≥ 4, the following methods will be carried out and re-evaluated until the patient leaves the PACU.

#### Postoperative treatment of special cases in PACU

(1) Postoperative pain: 0.03 mg/kg oxycodone or 0.01 µg/kg sufentanil.

(2) Postoperative agitation and delirium: dexmedetomidine 10 µg iv.

(3) Postoperative nausea and vomiting: 1 mg haloperidol.

Patients will be permitted to leave the PACU when their Aldrete score is ≥ 9.

All participants will be treated with postoperative patient-controlled intravenous analgesia. The analgesia protocol is sufentanil (250 µg) diluted in 250 mL 0.9% saline. The analgesic pump will administer a bolus dose of 4 mL, then 2 mL/ hour with a lockout interval of 8 min.

### Outcomes

#### Primary outcome

The primary outcome is the incidence of emergence agitation assessed via RASS in the PACU. Emergence agitation, evaluated every 10 min in the PACU, is defined as a RASS ≥ 2 at any time point.

RASS was developed by a multidisciplinary team at Virginia Commonwealth University in Richmond, VA, USA. It is a 10-point scale that can be rated briefly using 3 clearly defined steps, with discrete criteria for sedation and agitation levels [29]. The RASS has shown excellent interrater reliability in a broad range of adult medical and surgical patients and excellent validity compared with the visual analogue scale and selected sedation scales [30].

PACU nurses will assess RASS scores every 10 min in the PACU. If emergence agitation is diagnosed, patients will receive immediate attention and medication and be revaluated before leaving the PACU.

#### Secondary outcomes

Early postoperative delirium will be diagnosed on postoperative day 1 via the CAM, the most common diagnostic tool for delirium [31]. Delirium is diagnosed mainly depending on four characteristics: (1) acute fluctuation course, (2) attention disorder, (3) disordered thinking, and (4) changes in consciousness levels. Delirium can be diagnosed with both 1 and 2 and with either 3 or 4.

Postoperative pain immediately after awakening will be evaluated via the NRS [32]. The NRS is used to score patients’ postoperative pain degree and quantify their subjective postoperative pain using a number between 0 and 10. Patient’s pain statuses will be evaluated every 10 min in the PACU. For patients with NAS scores ≥ 4, analgesics will be given and re-evaluated.

Sleep quality measurement preoperative and postoperative day 1.

Nocturnal sleep quality will be evaluated on preoperative and postoperative day 1 using the RCSQ comprising five items: sleep depth, sleep latency, awakenings, return to sleep, and sleep quality. Each item is answered on a 100-mm visual analog scale. The scores range from 0 (worst possible sleep) to 100 (best possible sleep) [33].

Changes in levels of plasma melatonin, clock genes (BMAL1, PER2) and inflammatory factors (Il-6, TNF-A, CRP, PCT) will be detected preoperatively in the operating room and postoperatively in the PACU.

#### Other outcomes

Opioid consumption, related adverse reactions (e.g., respiratory depression, nausea, vomiting, pruritus, and urinary retention) during the operation, in the recovery room and on postoperative day 1 as well as postoperative surgical complications, ICU admission and hospital stay length will be measured.

### Analysis

Before locking the database, statistical analysis will be performed and confirmed by the principal investigator. Categorical variables will be reported as frequencies and percentages. Continuous variables conforming to a normal distribution will be presented as the mean ± standard error of the mean; other variables will be described as medians and interquartile range. The study will test whether the agitation incidences in groups T2 and T3 differ significantly from that of T1. A two-sided significance level of 0.025 (with Bonferroni’s adjustment for multiple comparisons) will be used for both comparisons.

Propensity score and propensity score-based inverse probability of treatment weighting (IPTW) will be used to adjust the covariates for reduction to a minimum selection bias and adjust for confounding patient characteristics. All thoracic surgeons from both centers will be surveyed to ensure all operation time selection factors are included as covariates in the model to further reduce potential confounding. The covariates include age, sex, BMI, ASA status, history of anesthesia and surgery, smoking, alcohol consumption, Charlson comorbidity index. Each variable will be analyzed by univariate logistic regression analysis. Variables reaching clinical significance (*p* < 0.10) will be entered into a multivariate logistic regression model as covariates.

A multivariate logistic regression model will be used to estimate patients’ propensity scores for operation time. After calculating the propensity scores, each patient will be weighted by the inverse of the probability of their treatment option (weight = 1/propensity score). After measuring the IPTW, the baseline characteristics will be well balanced in each group, and patients will be pooled for further analysis.

Emergence agitation incidence is defined as a RASS score ≥ 2 in the PACU at any time point. The emergence agitation incidence will be analyzed for T2 vs. T1 and T3 vs. T1 via the Pearson chi-squared test after IPTW adjustment. The risk of emergence agitation in weighted cohorts will be evaluated using the IPTW adjusted logistic regression model. The odds ratio for agitation with a 95% confidence interval will be calculated.

A linear mixed-model analysis will be used to examine the relationship between operation time and postoperative pain. Models for the linear mixed-effects models will be selected using the Akaike information criterion. The area under the curve (AUC) will be analyzed. Incidence of early postoperative delirium according to CAM score on postoperative day 1 will be analyzed in the same manner as the RASS score. RCSQ scores on preoperative and postoperative day 1 will be analyzed by covariance analysis after IPTW adjustment.

To explore the effects of circadian rhythms on emergence agitation in older individuals, logistic regression analysis will be used to investigate the association of clock rhythm with emergence agitation after adjustment with IPTW.

## Discussion

This paper describes a protocol to investigate the effects of circadian rhythms on emergence agitation and early postoperative delirium of older individuals undergoing thoracoscopic lung cancer surgery and explore the potential regulatory role of melatonin. To the best of our knowledge, the impact of different surgery times on agitation at times of emergence and in PACU exists. We aim to bridge this gap and seek new approaches to facilitate rapid recovery during the perioperative period.

### Strengths and limitations

A major strength of this study is that it is the first study to evaluate the effects of circadian rhythms on emergence agitation and early postoperative delirium of older people. Multiple evaluation methods such as RASS, CAM and NRS in the PACU are included in the current study. Clock gene and sleep assessment scales will be monitored for clock rhythms. This can provide a better and more comprehensive understanding of the connection between circadian rhythms and postoperative agitation and delirium in PACU.

This study will have its limitations. Although we will record RASS, NRS and CAM in PACU, it is important to acknowledge that such measurements are subjective, and more objective indicators may be necessary. Secondly, our study will be conducted after extubating in PACU, which might be insufficient for assessing postoperative cognition function. Follow-up will be needed to assess long-term postoperative cognitive effects.

#### Implications of this research

In the context of Enhanced Recovery After Surgery, the importance of improving the quality of perioperative rehabilitation quality of patients under multi-modal intervention has gained increasing recognition. We hope to get a new method for achieving rapid recovery during the perioperative period. By elucidating the mechanism through which circadian rhythms influence postoperative recovery, perioperative melatonin intervention may have the potential to reduce postoperative complications, shorten hospital stay and reduce the waste of medical resources.

## Data Availability

Participant data are stored in a ResMan Research Manager in accordance with the General Data Protection Regulations. Published data from this study cannot be traced to specific patients. The datasets generated and analyzed during the current study are not publicly available but are available from the corresponding author on reasonable request.

## Abbreviations

RASS: Richmond Agitation and Sedation Scale
PACU: post-anesthesia care unit
CAM: Confusion Assessment Method
NRS: numerical rating scale
BMAL1: brain and muscle Arnt-like protein 1
PER2: period circadian regulator 2
BMI: Body mass index
ASA: American Society of Anesthesiologists
RCSQ: Richards Campbell Sleep Questionnaire
IL − 6: interleukin-6
TNF − A: tumor necrosis factor-alpha
CRP: C-reactive protein
PCT: procalcitonin
SpO_2_: pulse oxygen saturation
EtCO_2_: end-tidal carbon dioxide
IPTW: inverse probability of treatment weighting
AUC: area under the curve
